# Supplementary figures and images for: Network Analysis of the Papaya Orchard Virome from Two Agroecological Regions of Chiapas, Mexico
Source: mSystems. 2020 Jan 14;5(1):e00423-19. doi: 10.1128/mSystems.00423-19 (PMC6967384; doi:10.1128/mSystems.00423-19)

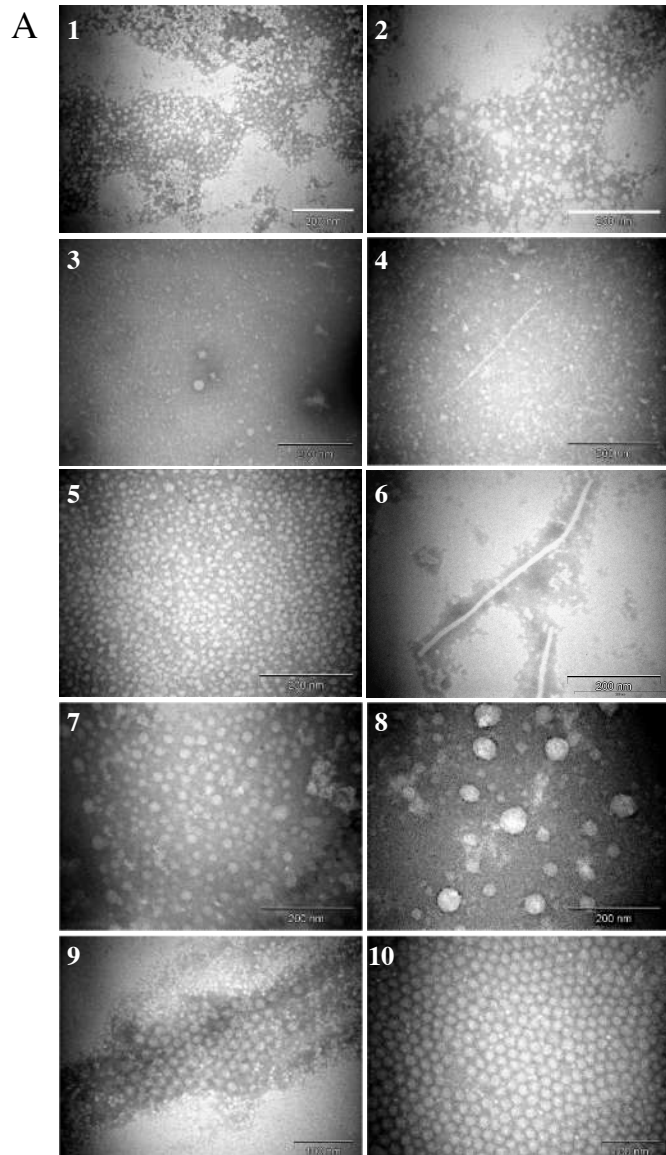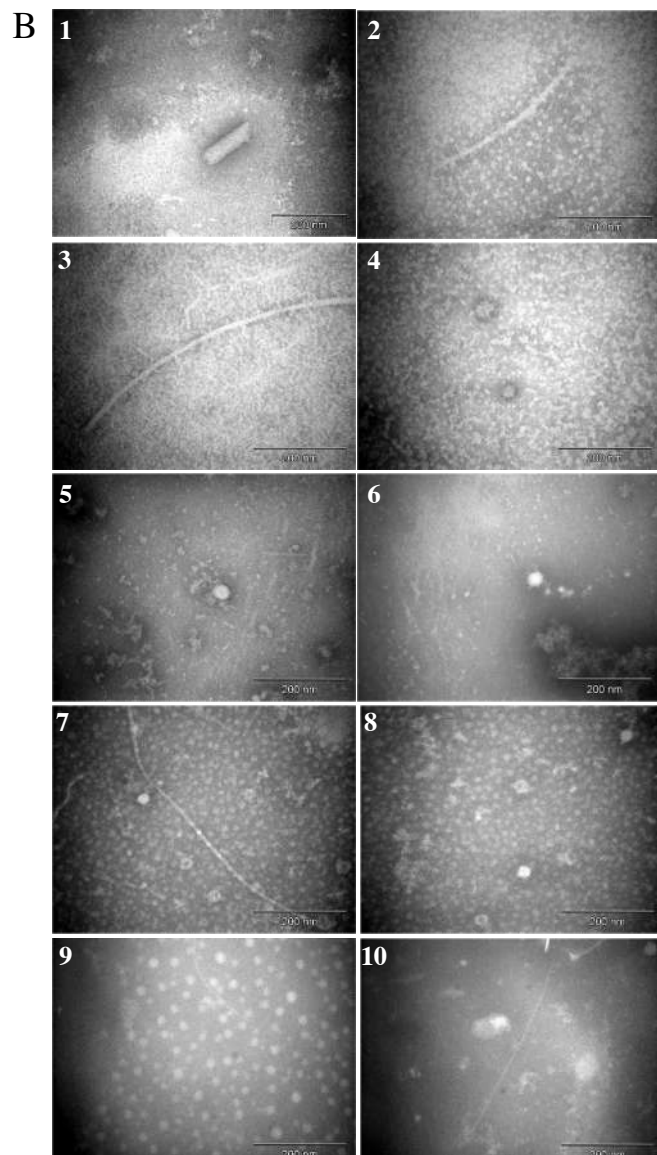

Supplement: FIG S1 [file mSystems.00423-19-sf001.pdf]
